# Effects of Intrauterine Infusion of Micronised Purified Flavonoid Fraction (MPFF) in Metritis-Diagnosed Dairy Cows Naturally Infected by *E. coli* during the Early Postpartum

**DOI:** 10.3390/vetsci9070362

**Published:** 2022-07-16

**Authors:** Miguel A. Gutiérrez-Reinoso, José B. Uquilla, Francisco A. Barona, Manuel E. Guano, Gloria N. Chicaiza, Manuel García-Herreros

**Affiliations:** 1 Facultad de Ciencias Agropecuarias y Recursos Naturales, Carrera de Medicina Veterinaria, Universidad Técnica de Cotopaxi (UTC), Latacunga 050150, Ecuador; manuel.guano3@utc.edu.ec (M.E.G.); noemig_1901@hotmail.com (G.N.C.); 2 Departamento de Ciencia Animal, Facultad de Ciencias Veterinarias, Universidad de Concepción (UdeC), Chillán 3780000, Chile; 3 Departamento de Asesoría Ganadera, La Holandesa SAS, Quito 170179, Ecuador; mvzjoseu@outlook.es; 4 Facultad de Ciencias de la Salud, Universidad de las Américas (UDLA), Quito 170125, Ecuador; fbarona@udlanet.ec; 5 Instituto Nacional de Investigação Agrária e Veterinária (INIAV), 2005-048 Santarém, Portugal

**Keywords:** MPFF, flavonoids, intrauterine infusion, metritis, postpartum, dairy cows

## Abstract

**Simple Summary:**

The occurrence of early metritis (EM) in dairy cattle causes serious economic losses. The treatment effectiveness for EM is highly controversial. Antibiotics administered by intrauterine infusions have been extensively used to control the growth of aerobic and anaerobic bacteria. Flavonoid glycosides (FGs) are polyphenolic naturally derived compounds considered to have many health-related properties such as antibiotic, anti-inflammatory, phlebotonic, and several vascular-protecting activities. Although FGs have been used for several therapies in humans, there is an increasing interest in investigating PGs for other therapeutic purposes. The objective of the present study was to investigate an alternative treatment for EM based on FG intrauterine infusions during the early postpartum period in dairy cows. FG administration contributed to the onset of uterine and cervix involution, bacteriological and immunological control, and fertility improvement associated with highly dose-specific responses during the early postpartum period in EM-positive dairy cattle.

**Abstract:**

The occurrence of metritis during the postpartum period causes serious economic losses in dairy cattle. The Micronised Purified Flavonoid Fraction (MPFF) is a polyphenolic flavonoid compound which is considered to have many health-related properties such as antibiotic, anti-inflammatory, phlebotonic, and several vascular-protecting activities. The aim was to evaluate the effects of a new strategic therapy for metritis based on MPFF intrauterine infusions during the early postpartum in dairy cows naturally infected by *Escherichia coli*. The clinical effects on reproductive anatomical structures and chronological involution dynamics were monitored until day 24 postpartum by ultrasonography. Moreover, uterine bacteriological and cytological (polymorphonuclear neutrophils; PMNs) profiles were analysed before and after MPFF infusion. The results showed that the success rate (% cure) at day 24 postpartum was improved significantly when using higher MPFF doses (*p* < 0.05). Moreover, MPFF treatment acutely diminished the size of the cervix and uterus and improved the involution process during the first 24 days (*p* < 0.05). The prevalence of pathogenic bacteria found in *in vitro* cultures was significantly variable (*p* < 0.01), as were the antibiotic sensitivity patterns. Pathogenic bacteria isolates decreased after MPFF applications in a dose–response fashion (*p* < 0.01), while isolates obtained from controls and low-dose-MPFF-treated animals were stable and similar (*p* > 0.05). The sensitivity patterns of pathogenic bacteria isolated in in vitro cultures from MPFF-treated animals were variable, although resistance to *E. coli*, *Staphylococcus aureus*, *Bacillus* spp., and coliforms was shown irrespective of the MPFF doses used. However, MPFF-treated cows showed a dose–response effect regarding PMN rates (*p* < 0.05). The calving-first service, calving–conception interval, and conception rate improved significantly from using higher MPFF doses (*p* < 0.05). In conclusion, this study shows that MPFF treatment differentially affects uterine involution, bacteriological profiles, cytological traits, and reproductive performance in metritis-positive dairy cows naturally infected by *E. coli*.

## 1. Introduction

Postpartum infertility and anoestrus are frequent phenomena in high-producing dairy cows [1,2]. Healthy dairy cattle on average attain complete cervical and uterine involution at 30–40 days postpartum [3]. This complex physiological process includes several stages such as uterine involution, a loss of tissue fluids, endometrial regeneration, ovarian cyclicity recovery, and pathogenic bacteria elimination [1,2]. Uterine wall oedema is gradually reduced from day 1 until it disappears by day 4–5 postpartum, while the cervix constricts from days 4–5 to 10 postpartum and then relaxes [3]. The most severe uterine involution occurs until day 3 postpartum and the earlier follicular activity is detected between days 4–5 and 10 post-calving [1]. Events involving uterine involution until day 12 postpartum include, among others, the necrosis of the caruncular stalk due to the decreasing blood supply and formation of the lochial discharge, which in normal conditions does not become purulent at any stage [4,5]. The hypothalamic–pituitary–ovarian–uterine axis recovers its functionality from days 10–12 to 24 postpartum and the uterine size decreases while its tone increases, coinciding with the onset of the first estrus. A subsequent involution continues slowly and by days 25–30, the uterine horns are almost equal in size [3,5]. The occurrence of early metritis (EM) disease in dairy cattle causes serious economic losses [1]. EM has been associated with an increased number of services per conception, lower conception rates, prolonged calving-to-conception intervals and higher culling rates [6]. Thus, the main consequences of EM are related to the negative impact on reproductive performance, diminishing productivity, and lowering life expectancy [7]. The EM (<21 d postpartum) is defined as a uterine inflammation of the endometrial mucosa and inner epithelial layers derived from an infection with no systemic disorder [8]. The risk of developing intrauterine infections increases drastically during the postpartum period due to several factors such as tissue damage derived from the parturition, dystocia, hypocalcaemia, prolapses or retained placenta, which delay uterine involution [9]. The endometrial epithelial layer is the first tissue barrier against bacterial infections. During the first days after parturition, the oestrogen-dependent blood supply enables the infiltration of leukocytes, neutrophils and lymphocytes, which play important roles in immune defence support in the uterine mucosa and inner epithelial layers [10]. Thus, postpartum oestrogens increase the number of uterine oxytocin receptors, which play a crucial role in the myometrial contractions, eliminating potential pathogens via uterine/vaginal discharges [11]. The PGF2 effects induce the luteolysis process and the advanced reduction in uterine size until day 24 postpartum. However, with the occurrence of EM, the pathogen-derived endotoxins disrupt ovarian function, modifying the normal release of GnRH/LH, altering the oestrogen levels (follicular and luteal phases), and increasing the incidence of follicular cysts and corpus luteum persistent cases [12]. A normal rectal temperature, enlarged-inflamed uterus with mucopurulent/watery content, abnormal uterine involution/thickness and vaginal discharges are just some of the most frequent clinical signs related to this reproductive disease, which may delay the endometrial regeneration or/and disrupt the cyclic ovarian function in the early postpartum cow [2]. There are several Gram-positive and Gram-negative microbial pathogens isolated from endometrial swabs or biopsy samples which can cause EM (e.g., *Fusobacterium*, *Escherichia*, *Prevotella*, *Campylobacter*, *Arcanobacterium*, *Trichomonas*, and α-haemolytic *Streptococcus*, among others) [13,14]. *E. coli* is one of the most prevalent bacteria involved in uterine diseases [8,15], and it can be detected as early as 1 to 3 days in milk (DIM) [16,17]. The actual *E. coli* endometrial pathogenesis, its therapeutic treatment by using alternative compounds, and its impact on fertility is poorly understood in dairy cattle. *E coli* lipopolysaccharides (LPS) provoke endometrial inflammation and trigger the production of pro-inflammatory cytokines, chemokines, major histocompatibility complexes and the attraction of immune cells in stromal tissue, affecting epithelial barriers [18,19,20,21]. In normal uterine involution conditions, the immune system resolves bacterial contamination; however, the presence of pathogenic species such as *E. coli* during the first weeks after parturition could trigger uterine diseases such as puerperal metritis [22]. Polymorphonuclear neutrophil leukocytes (PMNs) increase until days 10–12 postpartum and thereafter decline, whereas lymphocytes and macrophages increase until days 21–30 postpartum [23]. A cytological examination can give the percentage of PMNs and the neutrophil leukocyte/lymphocyte ratio [24,25,26]. PMN migration and phagocytic function are crucial factors for removing bacteria from the uterus [27]. It has been suggested that the occurrence of uterine diseases is associated with the elevated % PMNs in samples collected by flushing the uterine lumen or by endometrial Cytobrush [28]. The treatment effectiveness for EM is highly controversial. Antibiotics administered by intrauterine infusions such as tetracycline or third-generation cephalosporinns, among others, have been used extensively to control the growth of aerobic and anaerobic bacteria [29,30,31,32,33,34,35]. However, the results obtained from this and other antimicrobial agents were not entirely satisfactory due to clinical metritis, which may be triggered by other metabolic (negative energy balance), immunological (general and local) and management factors as well [36]. Flavonoid glycosides (FGs) are polyphenolic compounds sharing the same three-ringed-benzo-γ-pyrone (C6-C3-C6) molecular structure with hydroxyl (OH) groups attached. FGs are considered to have many health-related properties such as antibiotic, anti-inflammatory, phlebotonic and several vascular-protecting activities [37]. Diosmin (diosmetin 7-rhamnoglucoside; C_28_H_32_O_15_; MW: 608.6 Da) is a FG obtained from the flavonoid hesperidin (hesperitin 7-rhamnoglucoside; C_28_H_34_O_15_; MW: 610.6 Da). Diosmin ameliorates the symptoms related to chronic vascular inflammation and it is considered to have no effects on reproductive function or any other risks, such as embryo toxicity, transplacental migration or residues in milk, unlike other active principles such as oxytetracycline [38,39]. Micronised Purified Flavonoid Fraction (MPFF; diameter < 2 µm) increases the absorption and distribution of this throughout the body and it consists of 90% micronised diosmin, with 10% expressed as micronised hesperidin. MPFF is a well-known radical scavenger and has beneficial effects, through modulating key enzymes such as kinases, oxygenases and phospholipases, among others, increasing oxygen pressure, reducing prostaglandin synthesis, improving lymphatic drainage and reducing vascular hyperpermeability [40]. Although MPFF has been used for several therapies in humans, there is an increasing interest in investigating PGs for other therapeutic purposes in other mammals.

Thus, to gain insights into the effects of using non-conventional treatments, the main objective of the present study was to investigate a new treatment strategy for EM based on MPFF intrauterine infusions during the early postpartum period in dairy cows. For this purpose, the clinical effects on the reproductive tract’s anatomical structures, chronological involution dynamics, cytological characteristics and bacteriological profiles before and after MPFF treatment application were monitored until day 24 in metritis-diagnosed dairy cows naturally infected by *E. coli* during the early postpartum.

## 2. Materials and Methods

### 2.1. Reagents and Media

All reagents and media were purchased from Sigma (Sigma-Aldrich, St. Louis, MO, USA) unless otherwise stated, and they were used according to the manufacturer’s recommendations.

### 2.2. Environmental Conditions, Experimental Animals and Facilities

The present study was conducted on normal fertile Holstein–Friesian breed multiparous cows calved over a 3-year period (January 2015 to December 2017), which were kept on four commercial Ecuadorian dairy herds consisting of 150 ± 34 cows per farm located in Canton Mejía, Province Pichincha, Ecuador (coordinates: DMS latitude: 0°30′36.36″ S; DMS longitude: 78°34′11.28″ W; elevation: 3200 m.a.s.l.). The climate of this region is Cfb according to the classification of the Köppen–Geiger system, with temperatures ranging from 0 °C to 21 °C and a humidity of ~90%. At the time of study, the climate conditions posed no risk related to heat stress conditions, which could affect the reproductive performance or immunologic status of the animals, so all the studies were performed throughout the year. Cows (body condition score >2.5) were maintained in a pasture (*ad libitum* intake) and total mixed ration (TMR fed twice daily) consisting of corn silage, grass silage and a concentrate feeding regime with water *ad libitum*. All cows were milked two times daily (at 6 a.m. and at 6 p.m., controlled by a software milking system) and the annual average milk production was between 2500 and 3500 kg per cow/year. All individuals were identified and checked weekly by veterinary routine services to maintain the health of the herd (systemic diseases and increased somatic cell count, among others). During the study period, breeding management was carried out by the same veterinarianregarding cows’ behaviour, gynaecological signs of oestrus (three times daily), and clinical characteristics (reproductive health status). Artificial insemination (AI) was carried out using frozen–thawed semen doses from the same bull. Milk production parameters (volume, protein, fat and somatic cell count) were recorded by the Central Laboratory for Milk Recording (AGROCALIDAD, Tumbaco, Ecuador) after milk sampling once per week. Breeding management was not based on the use of hormones or any other active principle, and no animals were inseminated before 70 days postpartum. Cows that failed to come in heat were excluded from the study.

### 2.3. Experimental Design

**Figure 1:**
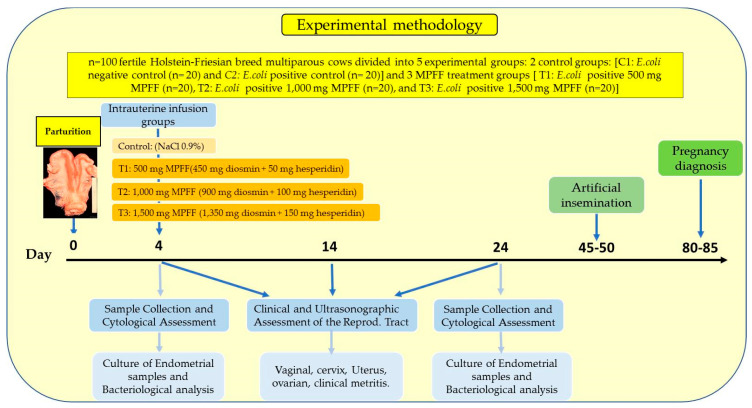
Experimental design map including control (2) groups and MPFF (3) treatment groups.

The experimental design (within-herd randomised controlled trial study; Figure 1) was carried out on the basis of postpartum clinical findings. For the first control group (negative control; *n* = 20), only postpartum healthy animals were enrolled (cows without symptoms of metritis—clean mucus), avoiding any periparturient disorder which could affect the reproductive performance, such as ketosis, mastitis, lameness, or displaced abomasum, as well as any other diagnosed pathologies related directly to the reproductive system (ovarian-, uterine- and cervix-related disorders). For the second control group (*E. coli* positive control), only cows displaying postpartum metritis episodes (cows with mild puerperal metritis—purulent lochia; *n* = 20; 4 d after calving) without any other disorder (to avoid any bias) were considered for the study [8]. Three additional treatment groups (*n* = 20 each, metritis and *E. coli*-positive) were enrolled in the study. Severe metritis implies an inflammation of the endometrium, and it was defined as the presence of purulent/bloody uterine discharge detectable in the vagina with a flaccid, non-retractable uterus (located in the abdomen), as well as a cervical diameter >75 mm and uterine horn diameter >60 mm in multiparous cows [41]. The reproductive activity was monitored during the months following treatments. The calving to conception interval, calving to first service interval and conception rate by day 85 postpartum were scored and compared among the control and experimental groups.

### 2.4. Experimental MPFF Intrauterine Infusion Treatments

For the experimental design (Figure 1), the cows were randomly divided into five experimental groups (negative control, positive control and three additional treatment groups (low dose: *n* = 20; medium dose: *n* = 20; high dose: *n* = 20)). The intrauterine infusion treatments were initiated on day 4 after calving and were performed on the same day. The first treatment group (low dose) received a single transcervically intrauterine infusion (day 0) of 20 mL (volume of the solution) of a compounded alcohol-free, water-based suspension of 500 mg micronised purified flavonoid fraction (MPFF Diosmina MK^®^; Lab. MK S.A.S., TQ Farma, Panama; intrauterine suspension of 450 mg diosmin + 50 mg hesperidin). The second (medium dose) and third (high dose) treatment groups were administered a single intrauterine infusion of 20 mL of the same suspension but containing 1000 mg (900 mg diosmin + 100 mg hesperidin) and 1500 mg (1350 mg diosmin + 150 mg hesperidin), respectively. Individuals belonging to the control group (negative and positive) received a transcervically intrauterine infusion of 20 mL of sterilised saline solution instead (NaCl 0.9%). Intrauterine infusion was applied with a disposable uterine catheter (53.5 cm Bovivet; Kruuse, Langeskov, Denmark) and a single-use 50 mL syringe. To evaluate the effect of each treatment on the reproductive tract involution, all cows underwent a clinical examination by intravaginal and transrectal palpation after thoroughly cleaning and disinfecting the perineal area with disinfectant solution, and were subjected to pre-treatment and post-treatment endometrial cytological evaluations.

### 2.5. Clinical and Ultrasonographic Reproductive Tract Evaluation

All cows were bred on observed estrus and pregnancy diagnosis was performed by transrectal ultrasonography (Aloka 500, 5 MHz linear transducer; Tokyo, Japan) of the uterus and its contents 35–40 days post-insemination. A total of 250 parturitions were evaluated for routine postpartum examination. All cows demonstrated normal cyclicity and they were multiparous (2nd to 5th parturition) without previous reproductive diseases (puerperal metritis, subclinical endometritis, clinical endometritis, retained foetal membranes, dystocia, caesarean section, puerperal mastitis, twins, stillbirth or abortion) based on their clinical data history. The involution of the uterus was estimated by transrectal ultrasonography according to its size (entire reproductive tract cervix + uterus). A postpartum ultrasonographic evaluation of the reproductive tract was carried out on days 4, 14, and 24 post-calving and the examination included the following parameters: lumen length, lumen width, and wall thickness for the uterus measurement and diameter and thickness for the cervix measurement. The examination included a manual vaginal examination (including any secretion from the vagina) and the transrectal palpation of the uterus. The functional states of the cervix (open or close), uterus (content) and ovarian structures (follicles and corpus luteum) were also examined. The confirmation of clinical metritis or healthy status was assessed by transrectal ultrasonography as well.

### 2.6. Sample Collection and Cytological Assessment

Bacteriological characteristics were assessed before and after MPFF intrauterine infusions throughout the experimental groups. All samples from the uterine epithelium were aseptically collected from each cow using a sterilised Cytobrush^®^ device (a cell collector probe designed to be inserted into the vagina and then passed through the cervix) for sampling the contents of the uterus (before and after intrauterine infusions), helped by a sterile, disposable vaginal speculum (Butler Animal Health Supply LLC, Dublin, OH, USA). A disposable transcervical catheter covered with a sanitary sheath was used to prevent brush contamination. The catheter was inserted into the vagina and pushed through the cervical canal into the uterus. Then, the brush was extruded from the catheter to take the samples from the endometrial mucosa. The samples were immediately inserted into the transport media for further isolation and analysis within 4 h after collection. Vaginal discharge was scored according to [42]. The criteria for selection were that they had a mucopurulent vulval discharge or any abnormality at rectal palpation confirmed by a cytological assessment of cervical mucosa. Differential cellular counts were conducted on air-dried and Giemsa-stained smears evaluated at high power under oil (×1000) and at least 10 fields were evaluated for each sample. To evaluate the effects of the MPFF infusions on the endometrium status and microflora, all cows were examined on day 4 (control before infusion) and day 24 after calving (last clinical examination was 20 days after the intrauterine infusion). Inflammation status was assessed based on the number of neutrophils per power field (PMNs/PF), including all leukocyte types and epithelial cells, but excluding erythrocytes, as described previously by Gilbert [43].

### 2.7. Culture of Endometrial Samples and Bacteriological Analysis

A microorganism culture and identification tests were processed according to standard microbiological techniques by using Blood agar and MacConkey agar media in the Central Laboratory for Microbiology Diagnosis from the National Agronomical Ministry (AGROCALIDAD, Quito, Ecuador). After inoculation, the plates were incubated aerobically and anaerobically at 37 °C for 18–24 h to promote the growth of bacteria, and then they were examined every 24 h for 7 days. Bacteriological procedures including determining the morphological characteristics of the colony, Gram staining, haemolysis and other standard tests such as citrate, urease catalase testing and biochemical confirmation (Micro-La Test, Pliva-Lachema Diagnostika, Brno, Czech Republic) were used for the culture identification of aerobic and facultative anaerobic bacteria. The number of bacterial colonies was recorded. Plates with more than three species were considered contaminated. Finally, the antimicrobial sensitivities of all isolated organisms were determined. In vitro antibiotic sensitivity tests from pure isolated colonies were carried out using culture antibiotic discs placed circularly on agar plates (amoxicillin, gentamicin, streptomycin, and ampicillin (10 μg); sulfamethoxazole–trimethoprim (25 μg); amoxicillin–clavulanic acid, cephalexin, cefazolin, and oxytetracycline (30 μg); diosmin–hesperidin (450 + 50 mg; 900 + 100 mg; 1350 + 150 mg)). The results (growth-inhibition zones) were interpreted to record the level of sensitivity after 24 h at 37 °C in aerobic/anaerobic conditions.

### 2.8. Statistical Analysis

Clinical data were retrieved from the cows in the different trial groups (MPFF-treated and non-MPFF-treated cows from both controls). ANOVA test (with variance being partitioned on the basis of between- and within-animal groups) was used to analyse the differences from the endometrial results obtained before and after MPFF treatments. When significant interactions were found, Duncan and Bonferroni tests were used (corrections for the between-animal group multiple comparisons). Moreover, Fisher’s exact test was applied to assess the differences in clinical and bacteriological findings. A Wilcoxon test/U Mann–Whitney tests were used for the comparison of pairs when an association between the nominal and ordinal variables was observed. A chi-square test was performed to analyse the differences in bacterial contamination among the groups. Endometrial cytologies on days 0, 10, and 20 of the experiment were compared using a Student’s *t* test. Reproductive parameters were compared using the Kruskal–Wallis test. The Shapiro–Wilk and Levene tests were used to evaluate the normality of the distribution of the continuous variables and the homogeneity of variances, respectively. Statistical analyses were performed to test the association between the different experimental groups. The discrete independent variables in all statistical models were the treatment group and cow. For all analyses, the individual cow was used as the experimental unit and the data were grouped taking into account the reproductive disease of the cows: normal-healthy versus uterine disease diagnosed-sick cows. Uterine disease and treatment status was included in the models as an independent variable and disease conditions were tested in the models as possible confounders. When an extreme heterogeneous variation among values was observed, data were logarithmically transformed prior to statistical analysis in order to remove heterogeneity of variance. Statistical analyses were carried out by using version 25.0 of the SPSS software (SPSS Inc., Chicago, IL, USA). For all analyses, values of *p* < 0.05 were considered significant.

## 3. Results

### 3.1. Effect of MPFF Intrauterine Infusion on the Success Rate (% Cure) in Metritis-Diagnosed Dairy Cows Naturally Infected by E. coli during the Early Postpartum Period

A total of 60 cases of clinical metritis were treated by using MPFF at different concentrations (low: 500 mg; medium: 1000 mg; high: 1500 mg). Twenty cases were involved for each treatment. Cases were considered resolved if the scoring system for the assessment of metritis reached 0 on day 20 (day 24 postpartum). The overall success rate (% cure) for all treatments was (24/60) 40%. Low, medium and high MPFF concentration treatments were successful in 15.0% (3/20), 45.0% (9/20), and 60.0% (12/20) of cows, respectively. There were significant differences among the success rates in low-, medium- and high-MPFF-dose treatments (*p* < 0.05; Table 1).

### 3.2. Effect of MPFF Intrauterine Treatments on Reproductive Tract Anatomical Dynamics in Metritis-Diagnosed Dairy Cows Naturally Infected by E. coli during the Early Postpartum Period 

The patterns of uterine and cervix dimension responses after administering intrauterine MPFF infusions in metritis-diagnosed cows were statistically different depending on the MPFF dose infused and the day after MPFF treatment assessed (*p* < 0.05; Table 2 and Table 3).

The dimension of anatomical structures such as the cervix and uterus (length, width and thickness) decreased on days 10 and 20 post-treatment, respectively, especially when the negative control and MPFF-treated cows at the medium (1000 mg) and the highest concentrations (1500 mg) were compared (*p* < 0.05). The negative control (healthy dairy cows) and medium- and high-concentration-treated groups (1000 mg and 1500 mg) showed significant dimensional differences in cervix diameter and uterus width when compared to the positive control and low-concentration-treated group (500 mg) on day 24 postpartum (*p* < 0.05). The uterine and cervix involution in MPFF-treated metritis-diagnosed groups had a consistently lower dimensional value on day 24, being significant at the highest dose of MPFF (1500 mg) (*p* < 0.05; Table 2 and Table 3).

This pattern was also followed by uterine and cervix dimensions in the negative control, but starting as early as day 14, being significant at the medium and highest doses (1000 mg and 1500 mg), respectively (Table 2 and Table 3; *p* < 0.05). In metritis-diagnosed cows, the uterus and cervix had their dimensions decreased significantly by day 14 only at the highest dose (1500 mg) (*p* < 0.05), which later decreased to close to the negative control’s response levels on day 24 postpartum (Table 2 and Table 3). Based on the diameter and number of follicular structures and ovary size response, no statistically significant differences irrespective of the controls or any metritis-treated among any control and/or treated groups along the study were observed (*p* > 0.05; data not shown).

### 3.3. Effect of MPFF Intrauterine Application on Uterine Bacteriological Profiles in Metritis-Diagnosed Dairy Cows during the Early Postpartum Period

Several bacterial genera were isolated from uterine samples obtained in control groups (negative and positive) and MPFF-treated cow groups (metritis-diagnosed individuals) on day 0 (day 4 postpartum) before MPFF application and after treatments on days 10 and 20 (days 14 and 24 postpartum, respectively). Pathogenic bacteria related to *E. coli*-produced metritis (*E. coli* alone or *E. coli* concomitant with other bacteria species) were only isolated from the metritis-diagnosed individuals (100%) compared to negative control individuals having no clinical signs (0%). More than twenty species (Gram-positive and Gram-negative) of aerobic, facultatively anaerobic and strictly anaerobic microorganisms were observed and classified according to their potential pathogenicity, including specific and non-specific genera/species. Moreover, bacterial frequency and growth scores were obtained from MPFF-treated group samples. More than 80% of the bacterial genera/species isolated (except for *E. coli*, which was present in all samples) were similar in metritis-diagnosed dairy cows on day 0 (day 4 postpartum) (*p* > 0.05; Table 4).

The most frequent isolates from both the control and MPFF-treated groups (metritis-diagnosed cows) derived from samples on day 4 postpartum belonged to the following genera/types: *Escherichia coli* (100%), *Staphylococcus epidermidis* (coag.−) (90.0%), Coliforms (≠spp.) (73.3%), *Lactobacillus* spp. (63.3%), *Bacillus* spp. (36.6%), *Staphylococcus aureus* (coag.+) (30.0%), *Streptococcus* spp. (26.6%), *Streptococcus* γ-haemolytic (20.0%), *Staphylococcus* spp. and *Enterococcus faecalis* (both 16.6%). Other isolated species were lower than these percentages (Table 4). From the total species/types isolated, nine were considered metritis-related pathogenic species (9/21; 42.8%). Different antibiotic sensitivity patterns of the pathogenic isolated bacteria obtained in in vitro cultures derived from intrauterine swab samples are shown in Table 5. In general, a higher antimicrobial sensitivity against Gram-negative bacteria was observed for antibiotics related to groups A to K. On the contrary, an antimicrobial sensitivity against Gram-positive bacteria was observed for antibiotics related to a wider spectrum (A to T antibiotic groups; see Table 5).

The isolate frequency did not differ significantly in the MPFF-treated compared to positive control group on day 0 (*p* > 0.05) (Table 6). After MPFF application (day 14 postpartum), the frequencies differed significantly when the positive control and low-dose-MPFF-treated groups were compared to high-dose-MPFF-treated cows (*p* < 0.05). Thus, the decreased frequencies and scores from pathogenic bacteria on days 10 and 20 MPFF post-treatment were associated with a decreased score for *Acinetobacter baumannii*, *Citrobacter freundii*, *Enterococcus faecalis*, *Streptococcus* γ-haemolytic and *Streptococcus viridans* together with maintained frequency or non-growth score changes for *Bacillus* spp., Coliforms (≠spp.), *Escherichia coli* and *Staphylococcus aureus* (coag.+) (Table 6). Thus, the occurrence of these bacteria was significantly higher in the positive control compared to high-dose-MPFF-treated cows (48/20 (2.40) versus 41/20 (2.05) for positive control and 55/20 (2.75) versus 30/20 (1.50) for high-dose-MPFF-treated group, respectively) when day 0 pre-treatment (day 4 postpartum) and day 20 post-treatment (Day 24 postpartum) scores were compared (*p* < 0.05). Different MPFF sensitivity patterns of the pathogenic isolated bacteria obtained in in vitro cultures derived from intrauterine swab samples are showed in Table 7. In general, the antimicrobial sensitivity increased as a dose-specific response. Thus, the higher the MPFF dose applied, the higher the antimicrobial sensitivity, especially for *Acinetobacter baumannii*, *Citrobacter freundii*, *Enterococcus faecalis*, *Streptococcus* γ-haemolytic and *Streptococcus viridians*. On the other hand, *Bacillus* spp., Coliforms (≠spp.), *Escherichia coli* and *Staphylococcus aureus* remained resistant even at the highest MPFF doses.

Out of the potential pathogenic bacteria isolated from metritis-affected cows, other non-pathogenic bacterial isolates were observed, of which *Pseudomonas aeuroginosa* and *Proteus mirabilis* were predominant (both 10.0%), followed by *Klebsiella oxytoca* and *Shigella flexneri* (both <10.0%) as Gram-negative bacteria, and *Staphylococcus epidermidis* (coag.−) (90.0%), *Lactobacillus* spp. (63.3%) and *Streptococcus* spp. (26.6%) were predominat as Gram-positive bacteria, followed by *Staphylococcus* spp. (16.6%), *Streptococcus agalactiae* (13.3%) and *Micrococcus* spp. (10.0%). Different bacterial genera/species/types, frequency per animal and range of scores are shown in Table 4. Finally, a positive correlation was observed between metritis-diagnosed cows and the percentage of pathogenic bacteria in the uterus irrespective of the MPFF-treated group studied (r = 0.59; *p* < 0.05).

### 3.4. Effects of MPFF Intrauterine Treatments on Uterine Cytological Characteristics in Metritis-Diagnosed Dairy Cows Naturally Infected by E. coli during the Early Postpartum Period

Cytological evaluation using uterine samples obtained by Cytobrush revealed differential PMN profiles depending on the sampling timepoint and the group analysed (Table 8). All of the uterine cytology slides were evaluated, revealing a high incidence of degenerative PMNs, especially in metritis-diagnosed cows on day 0 (day 4 postpartum), where 100% of cows showed a positive cytology for PMN (>18%). Thus, cytological evidence of inflammation was found in 100% of samples irrespective of the timepoint or group assessed, with the negative control being the lowest (8.2 ± 4.1% PMNs). The overall PMN (%) at the first Cytobrush collection (day 0) was ~20.0% (range from 18.8 *to* 22.1%) vs. ~36.0% at the last collection (day 20 after infusion) considering only metritis-diagnosed groups together (positive control and MPFF-treated cows). Thus, the % PMN values in metritis-diagnosed cows on day 4 postpartum were 18.8, 19.4, 22.1 and 21.5% for positive control and low-, medium-, and high-MPFF-dose-treated groups, respectively. No statistical differences were observed in % PMN among groups (*p* > 0.05). After 10 days post-infusion (day 14 postpartum) the PMN profile increased to 54.1, 50.9, 44.2, and 40.9% for positive control and low-, medium-, and high-MPFF-dose-treated groups, respectively.

On day 14 postpartum, significant differences were observed in % PMN when all control groups and MPFF-dose treatment groups were compared to day 4 (*p* < 0.05). However, on day 14 postpartum, no statistical differences were observed in % PMN between the positive control and low-MPFF-dose-treated or between medium- and high-MPFF-dose treated cows (*p* > 0.05). The PMN profiles on day 24 postpartum were 50.0, 41.2, 30.1, and 24.6% for positive control and low-, medium-, and high-MPFF-dose-treated cows, respectively. Thus, after 20 days post-infusion (day 24 postpartum), the PMNs profile decreased; however, statistical significant differences were only observed in % PMN when medium- and high-dose treatments were compared to day 14 postpartum (*p* < 0.05). No statistical differences were observed in % PMN regarding the positive control or low-MPFF-dose-treated group when compared to day 14 postpartum homologues (*p* > 0.05).

### 3.5. Effects of MPFF Intrauterine Application during the Early Postpartum Period on the Reproductive Performance Parameters in Metritis-Diagnosed Dairy Cows

Reproductive performance parameters were significantly different in negative control compared to positive control and MPFF-treated groups (metritis-diagnosed animals) (*p* < 0.05; Table 9). The calving–conception intervals (days open) in metritis-diagnosed cows were 136, 131, 120 and 117 for positive control, low-, medium-, and high-MPFF-dose treated cows, respectively. Significant differences were observed when positive control and low-MPFF-dose groups were compared to negative control or medium- and high-MPFF-dose-treated groups (*p* < 0.05). No differences were observed between the positive control and low MPFF-treated or between medium- and high-MPFF-dose-treated groups (*p* > 0.05). The days to first service (AI) in metritis-diagnosed cows were 112, 107, 95 and 93 for positive control and low-, medium- and high-MPFF-dose-treated cows, respectively. Significant differences were detected when positive control and low-MPFF-dose group were compared to negative control or medium- and high-MPFF-dose-treated groups (*p* < 0.05).

No differences were observed between positive control and low-MPFF-dose-treated or between medium- and high-MPFF-dose-treated cows (*p* > 0.05). Conception rates (%) were 25, 35, 50 and 60 in positive control and low-, medium- and high-MPFF-dose-treated cows, respectively. The positive effect of MPFF treatments on conception rates in metritis-diagnosed cows was considerably smaller than that observed for the negative control, especially when the negative control was compared to the positive control and low-MPFF-dose-treated cows (*p* < 0.05). Finally, differences were observed among all groups regarding the conception rate parameter (*p* < 0.05) (Table 9).

## 4. Discussion

In the present study, we reported for the first time the effects of intrauterine MPFF infusion in both healthy and metritis-diagnosed dairy cows as a mean to better understand the role of this active principle as a natural alternative for early uterine involution during the postpartum period and as treatment for *E. coli*-derived metritis-positive animals. Monitoring the dynamics of anatomical reproductive structures, cytological characteristics, and bacteriological profiles is crucial for the improvement of the reproductive performance in postpartum dairy cows [13,44]. However, metritis disease is not necessarily linked to only dairy cows because there is evidence of an important incidence in beef cows as well [45,46]. Early metritis prevention and treatment during the first days in the postpartum period may play a critical role in the future dairy cattle reproduction and productivity [47,48,49,50,51,52]. However, unlike for other health status cases [53,54,55], there is scarce research related to the use of natural alternative active principles to treat and prevent metritis in dairy cattle [56]. Diosmin, a well characterised drug with effects on the physiology of anti-inflammatory, phlebotonic and vascular-protecting activities, has led to the development of a wide range of clinical applications [57,58]. The MPFF effects in the short term were as expected in all experimental groups. Accordingly, the anti-inflammatory effects of other FGs decreased within weeks of administration, as reported for several species such as murine and humans [59,60].

In the negative control group (healthy individuals), the anatomical reproductive structures represented by the cervix and uterus decreased considerably after two weeks, while in metritis-diagnosed dairy cows, specifically in the positive control and low-MPFF-dose group, all structures gradually decreased slowly after 20 days of MPFF administration. As showed in the results, a significant dose–response effect was observed regarding uterine involution, with the higher-MPFF-dose group being the most influenced by MPFF-derived effects. Therefore, medium and high MPFF doses were required to activate the anti-inflammatory mechanisms in metritis-diagnosed cows. In vascular-protecting therapy treatments, there also seem to be differences in the FG pharmacodynamics [37,40]. Thus, lower MPFF doses are required in humans to treat general chronic vascular inflammation or other vascular diseases [61,62,63,64], while cows show enough local anti-inflammatory effects after the use of a relatively high MPFF dose (1500 mg). Although MPFF shows high levels of biosafety, comparable to those of other flavonoids [65,66,67,68], low doses (50–500 mg/day) are commonly used for therapeutic purposes to ameliorate chronic vascular inflammation in humans [61,62,69]. Thus, as shown in the present study, more studies based on MPFF dosing protocols will be needed to improve its effectiveness for metritis therapeutic purposes in dairy cattle.

Success values in metritis-diagnosed cows show that the % cure rates were variable depending on the dose used. This did not happen in healthy cows due to the unchanged healthy status throughout experiments. The overall success rate of all the treatments remained lower than 60% under the effect of MPFF in metritis-diagnosed cows; however, differences between the positive control and MPFF-treated animals were observed. Thus, there is evidence supporting higher MPFF doses involvement in health recovery success, probably due to the fact that lower doses were not as therapeutic in the present experimental design compared to any other FG doses, pathologies and species treated [37,40,59,60].

Even though uterine bacteriological profiles were pretty similar in metritis-diagnosed cows on day 0 (more than 50% of the bacterial genera/species isolated were similar in the positive control and MPFF-treated groups), cytological characteristics differed significantly. All cows had one uterine sample taken before MPFF treatments on day 4 postpartum, another one on day 14 and a third one on day 24 postpartum (both after MPFF treatment). Then, at 10 and 20 days post-infusion, all animals were evaluated again. In this specific time window, cytological characteristics in terms of % PMNs were elevated only in metritis-diagnosed cows, specifically in positive control and low-MPFF-dose-treated individuals, while bacteriological profiles (genera/species/types) were unaltered at a low dose and in the same groups. On the other hand, metritis-diagnosed cows had cytological characteristics in terms of % PMNs values above those of negative control animals 20 days after MPFF administration. The overall effects of MPFF as bactericide/bacteriostatic product against different genera/species were not conclusive, perhaps due to self-individual-specific sensitivity to the active principle, regardless of the dose or maybe due to *E. coli*-resistant strains [70]. In accordance with normal bacteriological profiles found in the negative control samples, the genera/species were unchanged during the experiments.

On the other hand, MPFF-treated cows did not show important variations among experimental groups in the calving–conception interval duration, specifically between positive control versus low-MPFF-dose group or between medium- versus high-MPFF-dose group. Furthermore, in order to see the effect of MPFF dose on fertility, days from calving to first service (AI) were estimated. Important differences were observed when positive control and low-MPFF-dose group were compared to medium- and high-MPFF-dose-treated animals, suggesting that the days from calving to first service (AI) were improved by higher MPFF doses in metritis-diagnosed dairy cows. The MPFF effects on the conception rates showed a similar pattern. This rate in MPFF-treated animals is consistent irrespective of the MPFF dose used, except for the low-MPFF-dose group. Accordingly, low/normal conception rates were observed in negative control animals. Interestingly, these were in the expected conception rate-range reported in the literature for dairy cattle [47,71], but MPFF-treated animals diagnosed as metritis positive on this work had lower conception rates than reported for healthy cows [72]. Accordingly, in the present study, high MPFF concentrations were applied as an efficient dose to accelerate uterine involution as well as fertility and reproductive performance in dairy cattle, which were similar when compared to other treatments [31,49,54,73,74]. The boost of cure rates after MPFF administration at high doses observed in metritis-diagnosed cows could hypothetically be traced back to either the MPFF dose, the very early diagnosis/treatment, or even both. Even though MPFF was administered as different intrauterine doses, it might specifically signal cervix receptors or even other receptors related to the ovary cycle as well [75]. This effect might not be related only to the reproductive anatomical dynamics but the immunological response-related signs would be affected as well due to the endogenous effects of MPFF-related molecules (diosmin/hesperidin), which would require crossing the uterine epithelium barrier in order to induce the desired effects to protect against potential infections and to increase the rhythm of the anatomical structure involution. As there is no evidence that MPFF can come across the uterine epithelium, the possibility remains that MPFF applied to metritis-diagnosed animals might have a local effect at a certain time window during the treatment that MPFF could take advantage of to come across the uterine and cervix epithelial mucous surface. Although important differences have been described among antibiotic applications as frequent traditional treatments in cows affected by metritis and endometritis [49,76,77,78], details about the fertility rates in metritis-affected dairy cows treated using MPFF and its relation to the dynamics of anatomical reproductive structures, cytological characteristics and bacteriological profile changes, particularly during the early postpartum period, are not available. The possibility that MPFF could leak in through the barrier at higher doses would explain, at least in part, the increased anti-inflammatory effects on tissues found in the present study via enzyme inhibition (e.g., via protein kinases or phosphodiesterases) and transcriptional factor modifications during the inflammation process (e.g., decreasing pro-inflammatory genes such as NF-κB, GATA-3, and STAT-6) [59,79,80]. Besides the possibility of the tissue unstable permeability related, it has been reported that flavonoids are potent antioxidants with the potential to attenuate tissue damage as well as several beneficial properties in vitro in inflammatory diseases [37]. Additionally, in humans and mice, innate immune cells produce pro-inflammatory cytokines and chemokines that attract lymphocytes and trigger an adaptive immune response such as several interleukins (IL-1 alpha, beta) and IL-6 [19,59]. Moreover, during the inflammatory immune response, reactive oxygen species (ROS), reactive nitrogen species (RNS) and different proteases are produced and they increase the tissue damage, fibrosis, and cell proliferation, contributing to the increase in inflammation-derived effects frequently found in diseases such as asthma, diabetes, CVD, neurodegenerative pathologies or cancer [81]. The molecular details of these effects through secluding biological barriers remain unknown and more studies are needed using different MPFF doses. On the other hand, the scavenger activity and the inhibition of free radical production derived from flavonoid effects probably take place via mediators such as prostaglandins, histamines, thromboxanes and leukotriene signalling through the inhibition of *PLA2*, *COX*, and *LOX*, but also by triggering local action tissue level, as has been shown previously [82]. During the modulation of the inflammatory process mediated by flavonoids, several immune cell types are affected by the inhibition of cell activation, maturation, signalling transduction and secretory processes, among other effects [37,40,60]. Furthermore, there is evidence of decreased cell proliferation and a lower release of pro-inflammatory cytokinesis involved in the inflammatory process mediated by flavonoids [37,40]. Thus, flavonoids can inhibit cell maturation by suppressing the expression of maturation markers (e.g., CD80/CD86) decreasing the proliferative response of CD4 + T cells and diminishing IL-1β, IL-4, IL-6, IL-8, IL-13, and IL-17 effects [59,83]. Furthermore, flavonoids can also bind to cytokine receptors and decrease signalling (e.g., by blocking IL-17 cytokine receptors) or even IgE receptors (e.g., FcεRI) [83]. MPFF might also directly or indirectly feed back to any other receptors located in different tissues. However, such feedback loops have yet to be described.

In the present work, MPFF’s effect in uterine tissues seems to be short-term contrary to the traditional long-term antibiotic effects. Dose-specific effects were clearly observed. Low-dose short-term unresponsiveness was probably due to an insufficient MPFF-related concentration, or even more applications would be needed during the experiments, as compared to other chemical therapeutic substances [32,33,34,35,48,84], together with a lower MPFF sensibility or higher dose requirements. More studies are needed on different application protocols and under a wider range of MPFF concentrations. Healthy cows from the negative control group, which had increased uterine involution values, the same as those associated with the use of high MPFF doses, also had better cytological characteristics, lower bacteriological profiles and a better reproductive performance. Similarly to healthy animals, within the metritis-diagnosed cows, the higher MPFF doses showed better results for all parameters studied, stimulating uterine self-renewal and involution, which may explain the effects of MPFF on the size of the anatomical structures and bacteriological profiles and the better rate of the reproductive performance compared to lower MPFF doses. However, metritis-diagnosed cows had more difficulties in achieving better recovering rates, so we suggest that the effects of MPFF were determined by the initial health status of the animals treated and more dosing protocols will be needed for specific purposes in dairy cattle as stated before for other therapeutic substances [32,33,34].

## 5. Conclusions

In conclusion, the present work suggests that MPFF administration contributes to the onset of uterine and cervix involution, bacteriological and immunological control, and fertility improvement associated with highly dose-specific responses during the early postpartum period in dairy cattle naturally infected by *E. coli*. MPFF concentrations will require adjustments in order to modulate and improve the uterus and cervix involution kinetics depending on the health status of the animals treated, with the aim of accelerating and improve the fertility potential. This study shows that the anti-inflammatory and bacteriostatic effects derived from administering MPFF in metritis-affected dairy cows naturally infected by *E. coli* leads to specific and differential responses, especially at high-concentration doses. This supports the fact that dose-specific protocols in the intrauterine administration of MPFF should be developed considering the particular sensitivity observed in the bacteriological and immunological responses and the conception rates among different experimental groups and MPFF doses. MPFF effects on reproductive tract involution were strongly evident at the highest doses in dairy cows. This represents a relevant factor to consider in order to improve the use of MPFF as an efficient natural alternative active principle seeking to accelerate uterine involution and preventing early metritis problems in dairy cows. More studies are needed to characterise the effect of MPFF, particularly dosing protocols required for specific therapeutic treatments in dairy cattle.

## Figures and Tables

**Table 1:** Effects of intrauterine infusion of Micronised Purified Flavonoid Fraction (MPFF; low, medium and high concentrations) on the success rate (% cure) at day 20 (day 24 postpartum) in metritis-diagnosed dairy cows naturally infected by *E. coli* during the early postpartum period.

Experimental Groups	(−) Control (*n* = 20; Healthy Non-Treated)	(+) Control (*n* = 20; Metritis Non-Treated)	Low Dose (*n* = 20; Metritis MPFF: 500 mg)	Medium Dose (*n* = 20; Metritis MPFF: 1000 mg)	High Dose (*n* = 20; Metritis MPFF: 1500 mg)
**Success Rate (%Cure; Day 20)** **(Day 24 Postpartum) Post-treatment**	0.0 ^A^	5.0 ^A^	15.0 ^B^	45.0 ^C^	60.0 ^D^
	(0/20)	(1/20)	(3/20)	(9/20)	(12/20)

Success rates (% cure) on day 20 after intrauterine infusion of Micronised Purified Flavonoid Fraction (MPFF; low, medium and high doses) in metritis-diagnosed dairy cows. Letters (A–D) within a row show statistical differences among controls/treatment groups (*p* < 0.05).

**Table 2:** Effects of intrauterine infusion of Micronised Purified Flavonoid Fraction (MPFF; low, medium and high concentrations) on uterine involution dynamics on days 0, 10, and 20 (days 4, 14, and 24 postpartum, respectively) in metritis-diagnosed dairy cows naturally infected by *E. coli* during the early postpartum period.

	Uterine Anatomical Parameters								
	Lumen Length (mm)			Lumen Width (mm)			Thickness (mm)		
	Day 0 (Day 4 Post-part) (Pre-Treat.)	Day 10 (Day 14 Post-Part.) (Post-Treat)	Day 20 (Day 24 Post-Part.) (Post-Treat)	Day 0 (Day 4 Post-Part.) (Pre-Treat)	Day 10 (Day 14 Post-Part.) (Post- Treat.)	Day 20 (Day 24 Post-Part) (Post-Treat)	Day 0 (Day 4 Post-Part) (Pre-Treat)	Day 10 (Day 14 Post-Part) (Post-Treat)	Day 20 (Day 24 Post-Part) (Post-Treat)
**(−) Control**	79.92 ± 0.30 ^Aa^	70.01 ± 0.21 ^Ba^	67.69 ± 0.15 ^Ba^	54.22 ± 3.91 ^Aa^	46.05 ± 3.16 ^ABa^	40.88 ± 2.92 ^Ba^	11.93 ± 0.90 ^Aa^	10.74 ± 0.46 ^Ba^	7.03 ± 0.31 ^Ca^
**(+) Control**	83.11 ± 0.33 ^Aa^	74.21 ± 0.23 ^ABa^	69.75 ± 0.19 ^Ba^	59.19 ± 4.56 ^Aa^	54.62 ± 3.84 ^ABa^	49.20 ± 2.87 ^Bb^	13.11 ± 0.95 ^Ab^	13.22 ± 0.42 ^Ab^	12.37 ± 0.69 ^Ab^
**Low Dose (500 mg)**	84.24 ± 0.40 ^Aa^	74.90 ± 0.32 ^ABa^	71.22 ± 0.28 ^Ba^	58.35 ± 4.61 ^Aa^	52.12 ± 2.98 ^ABa^	48.89 ± 3.73 ^Bb^	13.35 ± 1.33 ^Ab^	13.40 ± 0.67 ^Ab^	12.69 ± 0.32 ^Ab^
**Medium Dose (1000 mg)**	85.64 ± 0.39 ^Aa^	71.93 ± 0.28 ^Ba^	69.04 ± 0.19 ^Ba^	56.90 ± 4.39 ^Aa^	49.53 ± 3.81 ^ABa^	45.11 ± 3.19 ^Ba^	13.65 ± 1.41 ^Ab^	13.01 ± 0.52 ^Ab^	11.78 ± 0.46 ^Bb^
**High Dose (1500 mg)**	85.07 ± 0.45 ^Aa^	71.52 ± 0.22 ^Ba^	68.5 ± 0.24 ^Ba^	55.57 ± 5.03 ^Aa^	48.35 ± 4.88 ^ABa^	42.83 ± 4.77 ^Ba^	13.82 ± 1.25 ^Ab^	11.79 ± 0.61 ^Bc^	10.69 ± 0.30 ^Cc^

Anatomical measurements of uterine parameters on day 0 (before) and on days 10 and 20 after intrauterine infusion of Micronised Purified Flavonoid Fraction (MPFF; low, medium and high doses) in metritis-diagnosed dairy cows. Letters (A–C) within a row show statistical differences among timepoints (*p* < 0.05). Different letters in a column (a–c) show differences among controls/treatment groups (*p* < 0.05).

**Table 3:** Effects of intrauterine infusion of Micronised Purified Flavonoid Fraction (MPFF; low, medium and high concentrations) on cervical involution dynamics on days 0, 10 and 20 (days 4, 14 and 24 postpartum, respectively) in metritis-diagnosed dairy cows naturally infected by *E. coli* during the early postpartum period.

Experimental Groups	Cervical Anatomical Parameters					
	Diameter (mm)			Thickness (mm)		
	Day 0 (Day 4 Postpartum) (Pre-Treatment)	Day 10 (Day 14 Postpartum) (Post-Treatment)	Day 20 (Day 24 Postpartum) (Post-Treatment)	Day 0 (Day 4 Postpartum) (Pre-Treatment)	Day 10 (Day 14 Postpartum) (Post-Treatment)	Day 20 (Day 24 Postpartum) (Post-Treatment)
**(−) Control (*n* = 20; Healthy Non-Treated)**	44.25 ± 4.03 ^Aa^	42.56 ± 3.12 ^Aba^	35.89 ± 1.56 ^Ba^	14.10 ± 2.87 ^Aa^	9.99 ± 0.90 ^Aba^	6.78 ± 1.05 ^Ba^
**(+) Control (*n* = 20; Metritis Non-Treated)**	45.12 ± 3.55 ^Aa^	44.79 ± 3.32 ^Aa^	41.23 ± 1.81 ^Ab^	15.77 ± 3.03 ^Aa^	14.11 ± 0.82 ^Ab^	11.66 ± 1.58 ^Ab^
**Low Dose (*n* = 20; Metritis 500 mg)**	45.56 ± 4.67 ^Aa^	44.21 ± 3.78 ^Aa^	40.72 ± 1.45 ^Ab^	15.49 ± 2.99 ^Aa^	13.79 ± 1.97 ^Ab^	12.21 ± 1.90 ^Ab^
**Medium Dose (*n* = 20; Metritis 1000 mg)**	45.78 ± 3.37 ^Aa^	44.10 ± 2.67 ^Aba^	39.02 ± 1.63 ^Ba^	15.19 ± 1.83 ^Aa^	12.12 ± 1.29 ^ABab^	9.50 ± 1.10 ^Bab^
**High Dose (*n* = 20; Metritis 500 mg)**	45.46 ± 3.72 ^Aa^	43.87 ± 4.00 ^ABa^	37.90 ± 1.98 ^Ba^	15.88 ± 2.27 ^Aa^	11.89 ± 1.98 ^ABa^	8.85 ± 0.93 ^Bab^

Anatomical measurements of cervical parameters on day 0 (before) and on days 10 and 20 after intrauterine infusion of Micronised Purified Flavonoid Fraction (MPFF; low, medium and high doses) in metritis-diagnosed dairy cows. Letters (A–B) in a row show statistical differences among timepoints within the same parameter (*p* < 0.05). Different letters within a column (a–b) show differences among controls/treatment groups (*p* < 0.05).

**Table 4:** Identification and classification of Gram-negative and Gram-positive bacteria obtained in in vitro cultures derived from intrauterine swab samples (Cytobrush) based on their potential pathogenicity in producing metritis in dairy cows naturally infected by *E. coli* during the early postpartum period.

Bacterial Species/Type	Associated to Endometritis Cases/Considered Uterine Pathogen		Prevalence (% Total)	Prevalence (% Metritis +)
***GRAM*** ***−***				
***Acinetobacter baumannii*** *	Yes	Yes	2/120 (1.6%)	2/60 (3.3%)
***Coliforms*** (≠spp.) *	Yes	Yes	44/120 (36.6%)	44/60(73.3%)
***Citrobacter freundii*** *	Yes	Yes	6/120 (5.0%)	6/60 (10.0%)
***Escherichia coli*** *	Yes	Yes	60/120 (50%)	60/60 (100%)
***Klebsiella oxytoca***	No	No	4/120 (3.3%)	4/60 (6.6%)
***Pseudomonas aeuroginosa***	No	No	6/120 (5.0%)	6/60 (10.0%)
***Proteus mirabilis***	No	No	6/120 (5.0%)	6/60 (10.0%)
***Shigella flexneri***	No	No	2/120 (1.6%)	2/60 (3.3%)
***GRAM+***				
***Bacillus*** (≠spp.) *	Yes	Yes	11/60 (18.3%)	11/30 (36.6%)
***Clostridium*** **(≠spp.)**	No	No	2/60 (3.3%)	2/30 (6.6%)
***Enterococcus faecalis*** *	Yes	Yes	5/60 (8.3%)	5/30 (16.6%)
***Lactobacillus*** **(≠spp.)**	No	No	19/60 (31.6%)	19/30 (63.3%)
***Micrococcus*** **(≠spp.)**	No	No	3/60 (5.0%)	3/30 (10.0%)
***Staphylococcus*** **(≠spp.)**	No	No	5/60 (8.3%)	5/30 (16.6%)
***Staphylococcus aureus (coag.+)*** *	Yes	Yes	9/60 (15.0%)	9/30 (30.0%)
***Staphylococcus epidermidis (coag.−)***	No	No	27/60 (45.0%)	27/30 (90.0%)
***Staphylococcus hominis***	No	No	1/60 (1.6%)	1/30 (3.3%)
***Streptococcus*** **(≠spp.)**	No	No	8/60 (13.3%)	8/30 (26.6%)
***Streptococcus agalactiae*** *	No	No	4/60 (6.6%)	4/30 (13.3%)
***Streptococcus γ-haemolytic*** *	Yes	Yes	6/60 (10.0%)	6/30 (20.0%)
***Streptococcus viridans*** *	Yes	Yes	1/60 (1.6%)	1/30 (3.3%)
**Total**	998	908	90	90

* This microorganism has been associated to metritis cases and it should be considered as an uterine pathogen.

**Table 5:** Antibiotic sensitivity patterns of the pathogenic isolated bacteria obtained in in vitro cultures derived from intrauterine Cytobrush swab samples in metritis-diagnosed dairy cows naturally infected by *E. coli* during the early postpartum.

Bacterial Spp./Types	Antimicrobial Sensitivity Patterns																			
	A	B	C	D	E	F	G	H	I	J	K	L	M	N	O	P	Q	R	S	T
***GRAM−***																				
***Acinetob. baumannii***	++	++	++		++	-	++	+	-		++									
**Coliforms (** **≠** **spp.)**	++	++	++	++	++	+		-			++									
***Citrob. freundii***	++	++	++	++	+	+	+	+	-										+	
***E. coli***	++	++	++	++		+		+	-										+	
***GRAM+***																				
***Bacillus*** **(** **≠** **spp.)**	++	-		+	++	-		+	-		-			+	++		++			
***Enter. faecalis***						++			++	++	+	+						+	++	
***Staph. aureus*** **(c+)**	++	-		++	++	-	++	+	-		-			++	++		++	-		
***Strep.*** ***γ-hemolytic***	++	+		++	++	-		+	-				+		++					
***Strep. viridans***	++	+	++	+	+	-		-	-		+								-	
**Total HS (++)**	8	6	5	7	7	4	3	6	1	1	4	1	1	2	3	0	2	1	3	0
**Total R (−)**	0	2	0	0	0	5	0	2	7	0	2	0	0	0	0	0	0	1	1	0

Antimicrobial sensitivity patterns: (A) cephalosporins A (including cefuroxima, cefoxitina, ceftriaxona, cefotaxina/cefamandole, cefmetzole, ceftizoxima, cefepima, cefixima, ceftazidima, cefoperazona, ceftibutén, cefotetán, and ceftiofur); (B) quinolones (including norfloxacina, ofloxacina, enrofloxacina, levofloxacina, ácido nalidíxico, and gemifloxacina); (C) carbapenems (including imipenem and meropenem); (D) aminoglycosides (netilmicina, neomicina, gentamicina, amikacina, and sisomicina); (E) cephalosporins B (including cefalexina, cefazolina, cefalotina, cefaclor, cefadroxilo, and cefradina); (F) penicillins + β-lactamase inhibitors (amoxacilina + ácido clavulánico, and ampicilina + sulbactam); (G) cephalosporins C (cefotiam); H) sulfonamides + others (sulfonamides + trimethoprim); (I) penicillins I (amoxicilin); (J) glycopeptides (teicoplamina); (K) quinolones (norfloxacina, orfloxacina, enrofloxacina, moxifloxacina, and gatifloxacina); (L) glycopeptides II (vancomycin); (M) others (cloranfenicol and florfenicol); (N) tetracyclins (doxycliclina, oxitetracyclin, and tetraciclina); (O) macrolids (eritromicina and spiramycin); (P) others II (lincomicina, clindamicina, ácido fusídico, and polymyxin B); (Q) aminoglycosidos II (estreptomicina); (R) oxazolidinonas (linezolid); (S) quinolones II (ofloxacina, levofloxacina, and gemifloxacina); (T) penicillins II (ampicilin). Nomenclature: (−), resistant; (+), low/moderate sensitivity; (++), moderate/high sensitivity.

**Table 6:** Effects of intrauterine infusion of Micronised Purified Flavonoid Fraction (MPFF; low, medium and high concentrations) on the number of pathogenic bacterial isolates on days 0, 10 and 20 (days 4, 14 and 24 postpartum, respectively) in metritis-diagnosed dairy cows naturally infected by *E. coli* during the early postpartum period.

Experimental Groups (Number of Pathogenic Bacterial Isolates)					
Timepoints	(−) Control (*n* = 20; Healthy Non-Treated)	(+) Control (*n* = 20; Metritis Non-Treated)	Low Dose (*n* = 20; Metritis MPFF: 500 mg)	Medium Dose (*n* = 20; Metritis MPFF: 1000 mg)	High Dose (*n* = 20; Metritis MPFF: 1500 m)
**Day 0 (Day 4 Postpartum)** **(Pre-Treatment)**	0/20 (0.00) ^Aa^	48/20 (2.40) ^Ba^	52/20 (2.60) ^Ba^	50/20 (2.50) ^Ba^	55/20 (2.75) ^Ba^
**Day 10 (Day 14 Postpartum)** **(Post-treatment)**	0/20 (0.00) ^Aa^	50/20 (2.50) ^Ba^	46/20 (2.30) ^Ba^	40/20 (2.00) ^BCb^	35/20 (1.75) ^Cb^
**Day 20 (Day 24 Postpartum)** **(Post-treatment)**	0/20 (0.00) ^Aa^	41/20 (2.05) ^Ba^	43/20 (2.15) ^Ba^	34/20 (1.70) ^BCab^	30/20 (1.50) ^Cab^
**Total reduction (%) from Day 0 to Day 20**		7	9	16	25

Pathogenic bacterial isolates on day 0 (before) and on days 10 and 20 after intrauterine infusion of Micronised Purified Flavonoid Fraction (MPFF; low, medium and high doses) in metritis-diagnosed dairy cows. Letters (A–C) in a row show statistical differences among controls/treatment groups (*p* < 0.05). Different letters within a column (a–b) show differences among timepoints (*p* < 0.05).

**Table 7:** Micronised Purified Flavonoid Fraction (MPFF; low, medium and high concentrations) sensitivity patterns of the pathogenic isolated bacteria obtained in in vitro cultures derived from intrauterine swab samples (Cytobrush) in metritis-diagnosed dairy cows naturally infected by *E. coli* during the early postpartum period.

Bacterial Species/Type		Experimental Groups (Antimicrobial Sensitivity)		
	(+) Control (*n* = 20; Metritis Non-Treated)	Low Dose (*n* = 20; Metritis MPFF: 500 mg)	Medium Dose (*n* = 20; Metritis MPFF: 1000 mg)	High Dose (*n* = 20; Metritis MPFF:1500 mg)
***Acinetobacter baumannii*** *	−	+	++	+++
***Bacillus*** (≠spp.) *	−	−	−	+
***Citrobacter freundii*** *	−	+	++	+++
***Coliforms*** (≠spp.) *	−	−	−	+
***Enterococcus faecalis*** *	−	+	+	++
***Escherichia coli*** *	−	−	−	+
***Staphylococcus aureus (c+)*** *	−	−	−	+
Streptococcus *γ-haemolytic* *	−	+	++	+++
***Streptococcus viridans*** *	−	+	+	++

MPFF sensitivity patterns. Differences among controls/MPFF treatment groups. Nomenclature: (low, medium and high doses). (−), resistant; (+), low sensitivity; (++), moderate sensitivity; (+++), high sensitivity. * This microorganism has been associated to metritis cases and it should be considered as an uterine pathogen.

**Table 8:** Effects of intrauterine infusion of Micronised Purified Flavonoid Fraction (MPFF; low, medium and high concentrations) on the percentage (% PMNs) from intrauterine swab samples (Cytobrush) on days 0, 10 and 20 (days 4, 14 and 24 postpartum, respectively) in metritis-diagnosed dairy cows naturally infected by *E. coli* during the early postpartum period.

		Experimental Groups (% of Polymorphonuclears; % PMNs)			
Timepoints	(−) Control (*n* = 20; Healthy Non-Treated)	(+) Control (*n* = 20; Metritis Non-Treated)	Low Dose (*n* = 20; Metritis MPFF: 500 mg)	Medium Dose (*n* = 20; Metritis MPFF: 1000 mg)	High Dose (*n* = 20; Metritis MPFF: 1500 mg)
**Day 0 (Day 4 Postpartum)** **(Pre-Treatment)**	8.2 ± 4.1 ^Aa^	18.8 ± 9.2 ^Ba^	19.4 ± 11.6 ^Ba^	22.1 ± 10.1 ^Ba^	21.5 ± 13.6 ^Ba^
**Day 10 (Day 14 Postpartum)** **(Post-treatment)**	35.8 ± 9.7 ^Ab^	54.1 ± 14.2 ^Bb^	50.9 ± 18.7 ^Bb^	44.2 ± 20.1 ^ABb^	40.9 ± 16.8 ^Ab^
**Day 20 (Day 24 Postpartum)** **(Post-treatment)**	16.1 ± 6.0 ^Aa^	50.0 ± 19.6 ^Bb^	41.2 ± 14.4 ^Bb^	30.1 ± 16.5 ^Ca^	24.6 ± 18.1 ^ACa^
**Total reduction (%) from Day 10 to Day 20**	19.7 ^A^	5.5 ^B^	9.7 ^B^	14.1 ^AB^	16.3 ^AB^

Polymorphonuclear neutrophils percentage (PMNs%). PMNs% in endometrial samples collected on days 0, 10 and 20 after intrauterine infusion of Micronised Purified Flavonoid Fraction (MPFF; low, medium and high doses) in metritis-diagnosed dairy cows. Letters (A–C) within a row show statistical differences among controls/treatment groups (*p* < 0.05). Different letters within a column (a–b) show differences among timepoints (*p* < 0.05).

**Table 9:** Effects of intrauterine infusion of Micronised Purified Flavonoid Fraction (MPFF; low, medium and high concentrations) during the early postpartum period on the reproductive performance parameters in metritis-diagnosed dairy cows naturally infected by *E. coli*.

Reproductive Performance Parameters	Experimental Groups				
	(−) Control (*n* = 20; Healthy Non-Treated)	(+) Control (*n* = 20; Metritis Non-Treated)	Low Dose (*n* = 20; Metritis MPFF: 500 mg)	Medium Dose (*n* = 20; Metritis MPFF: 1000 mg)	High Dose (*n* = 20; Metritis MPFF: 1500 mg)
**Calving–Conception Interval** **(CCI; Days)**	106 ± 29 ^A^	136 ± 41 ^B^	131 ± 45 ^B^	120 ± 40 ^C^	117 ± 37 ^C^
**Calving to first service Interval** **(CFSI; Days)**	85 ± 21 ^A^	112 ± 34 ^B^	107 ± 39 ^B^	95 ± 44 ^C^	93 ± 40 ^AC^
**Conception Rate (%)**	75.0 ^A^	25.0 ^B^	35.0 ^C^	50.0 ^D^	60.0 ^E^
	(15/20)	(5/20)	(7/20)	(10/20)	(12/20)

Reproductive performance parameters after intrauterine infusion of Micronised Purified Flavonoid Fraction (MPFF; low, medium and high doses) in metritis-diagnosed dairy cows. Letters (A–E) in a row show statistical differences among controls/treatment groups (*p* < 0.05).

## Data Availability

Not applicable.
